# Comprehensive coronary computed tomography angiography assessment in high-altitude diabetic populations: integrating plaque characteristics, pericoronary inflammation, and coronary function

**DOI:** 10.3389/fcvm.2026.1886360

**Published:** 2026-10-09

**Authors:** Xin Fang, Shuang Li, Zhetao Wang, Junyan Zhang, Jia Song, Bing Wu, Kang Li, Kaiyue Diao

**Affiliations:** 1 West China Hospital, Sichuan University, Chengdu, China; 2 BioMedical Engineering and Imaging Institute, Icahn School of Medicine at Mount Sinai, New York, NY, United States

**Keywords:** CCTA, coronary plaque, diabetes mellitus, high altitude, pericoronary adipose tissue

## Abstract

**Background:**

Coronary computed tomography angiography (CCTA) provides complementary anatomical, inflammatory, calcific, and functional biomarkers, but their interpretation under chronic high-altitude exposure remains uncertain. We characterized CCTA phenotypes according to altitude and type 2 diabetes and explored associations with major adverse cardiovascular events (MACE).

**Methods:**

This retrospective matched cohort with prospective outcome ascertainment included adults undergoing clinically indicated CCTA for suspected coronary artery disease. Participants were classified by high-altitude residence (≥2,500 m for ≥5 years) or low-altitude residence (<500 m) and by diabetes status, forming four groups. Propensity score matching was performed separately within diabetes strata using age, sex, body mass index, and hypertension, yielding 174 participants in 87 matched pairs. CCTA assessment included pericoronary adipose tissue (PCAT) attenuation, plaque characteristics, coronary artery calcium (CAC), coronary stenosis, and vessel-specific CT-derived fractional flow reserve (CT-FFR). Primary models evaluated altitude, diabetes, and their interaction with adjustment for age, sex, body mass index, and hypertension. Associations with MACE were explored using univariable Cox models among participants with follow-up.

**Results:**

In primary covariate-adjusted analyses, diabetes was associated with higher PCAT attenuation, CAC, total plaque volume, plaque burden, and maximum stenosis and with lower left anterior descending (LAD) CT-FFR (all q ≤ 0.018). Compared with the low-altitude Han group, the high-altitude Tibetan group had higher LAD and right coronary artery CT-FFR and lower CAC and plaque burden (all q ≤ 0.037). Among 13 interaction tests, only the altitude × diabetes interaction for LAD-PCAT remained significant after false discovery rate correction (−6.62 HU; 95% CI, −10.32 to −2.91; raw *P* < 0.001; q = 0.008). Among 119 participants with follow-up, 20 MACE occurred over a median of 28 months. Exploratory univariable analyses showed large but imprecise associations of diabetes and high-risk plaque with MACE, whereas altitude was not statistically associated with MACE (*P* = 0.557).

**Conclusion:**

Diabetes was associated with a more adverse coronary CCTA phenotype, whereas the high-altitude Tibetan group showed lower CAC and plaque burden and higher vessel-specific CT-FFR than the low-altitude Han group. The association between diabetes and LAD-PCAT attenuation differed between these groups. These findings warrant validation in larger, ethnically comparable longitudinal cohorts.

## Introduction

According to the 11th edition of the International Diabetes Federation Diabetes Atlas, an estimated 589 million adults aged 20 to 79 years worldwide had diabetes in 2024 (11.11%), and this number is projected to reach 853 million by 2050 (12.96%) (1). People with diabetes have a twofold to fourfold higher risk of cardiovascular events (2, 3). Despite advances in treatment, cardiovascular complications account for 50% to 80% of diabetes-related mortality (4, 5), highlighting the need for improved cardiovascular risk stratification. Coronary computed tomography angiography (CCTA) enables comprehensive noninvasive assessment of coronary atherosclerosis, including plaque burden and high-risk plaque features (6, 7), pericoronary adipose tissue (PCAT) attenuation as a marker of coronary inflammation (8–10), coronary artery calcium (CAC) scoring (11), and CT-derived fractional flow reserve (CT-FFR) for functional assessment (12). These CCTA-derived imaging biomarkers provide prognostic information beyond traditional risk factors in patients with diabetes (13). However, most validation studies have been conducted in sea-level populations, leaving their applicability to individuals chronically exposed to high-altitude hypobaric hypoxia uncertain.

Approximately 140 million people worldwide live at elevations of ≥2,500 m and are chronically exposed to hypobaric hypoxia (14). Some epidemiological studies have reported an inverse association between altitude and coronary heart disease mortality, including an estimated 22% reduction in mortality for each 1,000 m increase in elevation (15, 16). However, evidence regarding the relationship between altitude and diabetes has been inconsistent. A lower prevalence of diabetes has been reported in high-altitude populations in the United States (17), whereas greater dysglycemia has been observed among populations living above 3,500 m in another setting (18). Whether chronic high-altitude exposure is associated with differences in CCTA-derived coronary imaging biomarkers in individuals with and without diabetes remains unclear.

Therefore, we aimed to characterize CCTA-derived coronary imaging biomarkers, including plaque characteristics, PCAT attenuation, CAC, and CT-FFR, in participants with and without diabetes residing at high or low altitude. As a secondary aim, we explored the occurrence of major adverse cardiovascular events across the four altitude and diabetes groups and examined the associations of clinical and imaging variables with cardiovascular outcomes.

## Methods

### Study design and population

This retrospective matched cohort with prospective outcome ascertainment was conducted at West China Hospital, Sichuan University. Baseline clinical and imaging data were extracted retrospectively, and outcomes were subsequently ascertained through medical-record review and structured telephone follow-up. The study protocol was approved by the Institutional Ethics Committee of West China Hospital (Approval No. 20231529). The approved protocol covered structured telephone follow-up and outcome ascertainment and waived the requirement for written informed consent. The study flowchart is presented in Figure 1.

**Figure 1:**
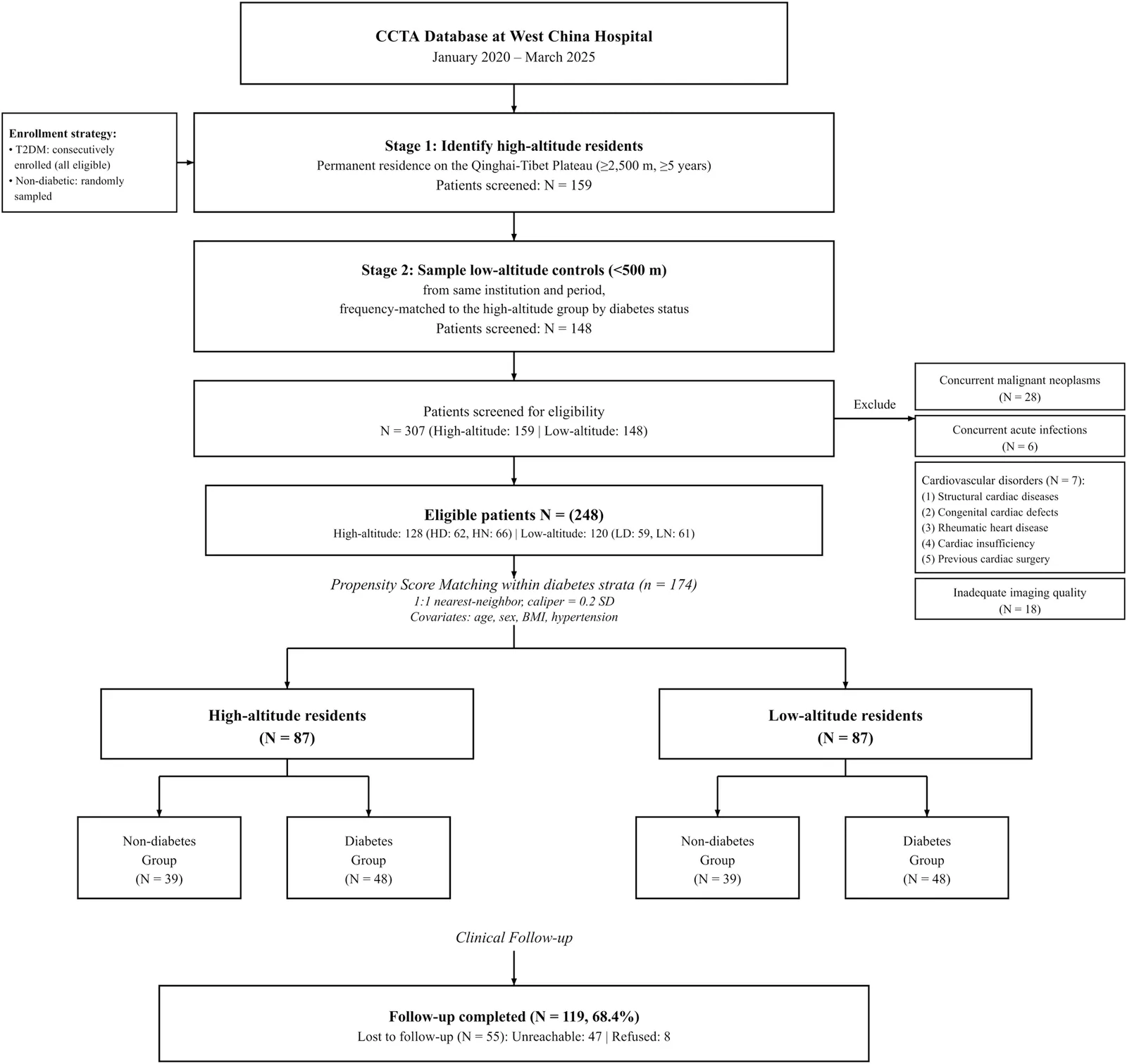
Patient selection and cohort construction. The source cohort comprised 248 eligible participants; propensity-score matching was performed separately within diabetes strata using age, sex, body mass index, and hypertension, yielding 174 participants (87 high-altitude and 87 low-altitude), of whom 119 had follow-up. BMI, body mass index; CCTA, coronary computed tomography angiography; HD, high-altitude diabetes; HN, high-altitude non-diabetes; LD, low-altitude diabetes; LN, low-altitude non-diabetes; PSM, propensity score matching; T2DM, type 2 diabetes mellitus.

Patients who underwent clinically indicated CCTA for clinician-suspected coronary artery disease between January 2020 and March 2025 were identified from the institutional imaging database. Clinician-suspected coronary artery disease was defined as referral for CCTA after assessment by the referring clinician; this assessment could be prompted by chest pain or chest discomfort, exertional dyspnea, cardiovascular risk factors, and/or prior test findings (19). High-altitude residence was defined as permanent habitation on the Qinghai-Tibet Plateau at ≥2,500 meters above sea level (20), with a minimum duration of 5 years to represent prolonged exposure to chronic hypobaric hypoxia (21). Chen et al. compared recent migrants (3–12 months) with long-term residents (5–10 years); accordingly, the 5-year threshold was used here as an operational criterion for prolonged residence rather than a universal biological cutoff (21). Among high-altitude residents, all patients with type 2 diabetes mellitus (T2DM) meeting eligibility criteria were consecutively enrolled, while non-diabetic patients were randomly sampled from the same period to achieve comparable group sizes. T2DM was defined by a prior established diagnosis or current glucose-lowering treatment; when diagnostic laboratory data were available, American Diabetes Association laboratory criteria were also applied (22). Diabetes status was not assigned solely on the basis of a single fasting or postprandial glucose measurement at enrollment. Low-altitude controls were randomly sampled from patients undergoing CCTA at the same institution during the same period and were frequency-matched to the high-altitude group by diabetes status. Low-altitude residence was defined as habitation at <500 meters above sea level. All high-altitude participants were Tibetan, whereas all low-altitude participants were Han. Residence category was determined from outpatient records, electronic medical records, and telephone confirmation. For an exploratory analysis within the high-altitude group, participants in the final matched cohort were classified into residential-altitude subgroups of 2,500–3,999 m and ≥4,000 m.

Inclusion criteria for both groups were: (1) age ≥18 years; (2) permanent residence ≥5 years at the respective altitude. Exclusion criteria included: (1) concurrent malignant neoplasms; (2) active systemic infections; (3) structural heart disease, congenital heart defects, rheumatic heart disease, heart failure, or prior cardiac surgery; and (4) inadequate CCTA image quality precluding quantitative analysis (Figure 1).

### Propensity score matching

To further reduce measured confounding between altitude groups, propensity score matching (PSM) was performed separately within the diabetes and non-diabetes strata. Within each stratum, the probability of high-altitude residence was estimated by logistic regression using age, sex, body mass index, and hypertension. One-to-one nearest-neighbor matching without replacement used a caliper of 0.2 standard deviations of the propensity score. The source cohort comprised 248 participants and yielded 174 matched participants in 87 pairs. Balance was assessed using standardized mean differences (SMDs), with |SMD|<0.10 used as the reference criterion. The overall study design and principal findings are summarized in Figure 2.

**Figure 2:**
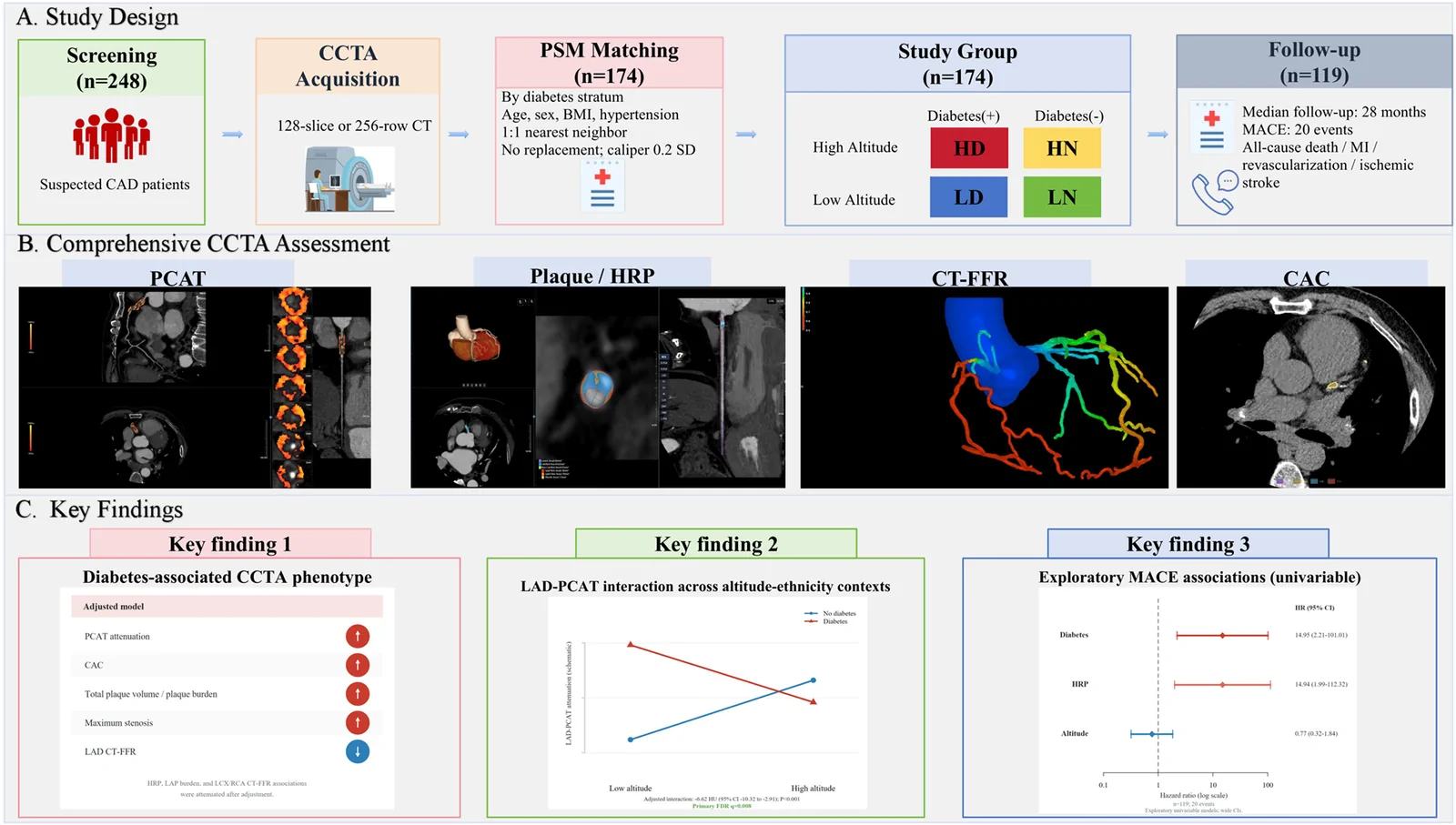
Study overview and principal findings. **(A)** The source cohort (*n* = 248) underwent propensity score matching separately within diabetes strata using age, sex, body mass index, and hypertension, yielding 174 participants (87 pairs); follow-up was available for 119 participants, with 20 MACE. **(B)** Representative CCTA assessment domains. **(C)** Schematic summary of diabetes-associated CCTA phenotypes, the altitude × diabetes interaction for LAD-PCAT, and the exploratory MACE analysis; detailed estimates and significance tests are presented in the corresponding Results sections, tables, and figures. BMI, body mass index; CAC, coronary artery calcium; CAD, coronary artery disease; CCTA, coronary computed tomography angiography; CT-FFR, CCTA-derived fractional flow reserve; CV, cardiovascular; HD, high-altitude diabetes; HN, high-altitude non-diabetes; HRP, high-risk plaque; LAD, left anterior descending artery; LD, low-altitude diabetes; LN, low-altitude non-diabetes; MACE, major adverse cardiovascular events; MI, myocardial infarction; PCAT, pericoronary adipose tissue attenuation; PSM, propensity score matching; Revasc, revascularization; SD, standard deviation.

### Follow-up and outcome assessment

Follow-up began on the date of the baseline CCTA examination and continued until the first MACE or the last documented contact; participants without MACE were censored at their last documented contact. Outcomes were ascertained through electronic medical-record review and structured telephone interviews after baseline data extraction. MACE was defined as the composite of all-cause death, acute myocardial infarction diagnosed according to the Fourth Universal Definition of Myocardial Infarction, refractory angina requiring coronary revascularization, or ischemic stroke (23). All endpoint events were independently adjudicated by two cardiologists blinded to imaging findings, with disagreements resolved by consensus.

### CCTA acquisition

All CCTA examinations were acquired at West China Hospital, a low-altitude imaging site, with patients supine on either a dual-source 128-slice CT scanner (SOMATOM Definition Flash; Siemens Healthineers, Forchheim, Germany; 100 kVp) or a 256-row wide-detector CT scanner (Revolution CT; GE HealthCare, Waukesha, WI, USA; 120 kVp). Prospective electrocardiographic gating, automatic tube-current modulation, weight-based intravenous iomeprol (400 mgI/mL), bolus tracking, and 0.625 mm reconstruction were used. Scanner type and tube-voltage distributions across the four study groups are reported in Supplementary Table S1.

### CCTA image analysis

All CCTA datasets were processed using the automated analysis platform (uAI Discover-CoronaryCTA, Version R001; United Imaging Healthcare, Shanghai, China) (24). The AI system automatically generated plaque, PCAT, CAC, and CT-FFR measurements. Two trained readers (X.F. and S.L.), blinded to clinical data and group allocation, reviewed the automated outputs for technical quality, with a senior cardiovascular radiologist (K.D., with 10 years of experience in cardiac CT imaging) consulted when output quality was uncertain. No AI-generated quantitative result was modified during this review. Because no manual repeat measurements were performed, reader-based intraobserver and interobserver reproducibility statistics were not available.

Coronary arteries were evaluated using the modified 18-segment model of the Society of Cardiovascular Computed Tomography (SCCT), with segments ≥1.5 mm in diameter included in the analysis (25). Coronary stenosis was graded according to the Coronary Artery Disease Reporting and Data System 2.0 (CAD-RADS 2.0) (26), and maximum per-patient stenosis was determined as the most severe luminal narrowing across all analyzed segments. For analysis, CAD-RADS categories were grouped into three clinically relevant strata: no CAD (CAD-RADS 0), non-obstructive CAD (CAD-RADS 1–2, representing 1–49% stenosis), and obstructive CAD (CAD-RADS ≥3, representing ≥50% stenosis).

Quantitative plaque analysis was performed for each coronary segment using automated tracing of the vessel wall and lumen boundaries. Total plaque volume (mm^3^) was calculated as the sum of all identified plaques across the left main(LM), left anterior descending artery(LAD), left circumflex artery (LCX), and right coronary artery (RCA). Plaque composition was classified based on CT attenuation as non-calcified, partially calcified (mixed), or calcified. Plaque burden (%) was defined as total plaque volume divided by total vessel volume × 100%. Low-attenuation plaque (LAP) burden was calculated as LAP volume divided by total plaque volume × 100%. High-risk plaque (HRP) features were defined in accordance with CAD-RADS 2.0 as follows: (1) positive remodeling (remodeling index >1.1); (2) low-attenuation plaque [CT attenuation <30 Hounsfield units (HU)]; (3) spotty calcification (<3 mm in any direction surrounded by non-calcified plaque); and (4) napkin-ring sign (ring-like higher attenuation surrounding a low-attenuation core). Plaques exhibiting ≥2 high-risk features were classified as vulnerable (CAD-RADS modifier V) (26).

CAC was quantified from non-contrast CT images using the Agatston method (27). Total and segment-specific CAC scores were calculated for the left main, LAD, LCX, and RCA.

PCAT attenuation was calculated continuously by the automated platform in the proximal 40 mm of the RCA, LAD, and LCX. The perivascular radial distance equaled the local vessel diameter, and voxels from −190 to −30 HU were included (8).

CT-FFR was computed from the standard CCTA datasets using the validated machine-learning workflow, with vessel-specific LAD, LCX, and RCA values; ≤0.80 was used as the conventional hemodynamic reference (28). Because the source images were acquired at low-altitude West China Hospital, CT-FFR reflects physiology derived under the examination conditions rather than a direct measurement during hypobaric hypoxia.

### Statistical analysis

Continuous variables were summarized as mean ± SD or median [interquartile range] after assessment with the Shapiro–Wilk test, and categorical variables as *n* (%). Medication use was reported as n/N among participants with nonmissing records, with class-specific missingness; missing values were neither treated as nonuse nor imputed, and medications were excluded from modeling. Hemoglobin and oxygen saturation were summarized descriptively, and oxygen saturation was not included in adjusted models because it was available for only 31 participants.

Primary imaging models included altitude, diabetes, their interaction, age, sex, body mass index, and hypertension. Continuous outcomes used linear regression with HC1 variance clustered by matched pair, HRP used pair-clustered logistic regression, and three-level CAD severity used multinomial logistic regression with 500 matched-pair cluster bootstrap replicates because the proportional-odds assumption was not met. Total CAC was modeled as log2(CAC + 1), and total plaque volume, LAP burden, and plaque burden as log(x + 1). Lipid profile, HbA1c, fasting glucose, medication use, and diabetes duration were not included in adjustment. Within the high-altitude group, 2,500–3,999 m and ≥4,000 m subgroups were compared using Welch's *t* test or the Mann–Whitney U test for continuous variables and Fisher's exact test for categorical variables; these comparisons were exploratory. For the scanner-adjusted LAD-PCAT sensitivity analysis, scanner type was added to the primary model.

Because only 20 MACE occurred, survival analyses were exploratory and univariable. Kaplan–Meier curves were compared by log-rank tests, and individual associations were estimated with matched-pair-robust Cox models; proportional hazards were assessed using scaled Schoenfeld residuals. CT-FFR was analyzed separately by vessel per 0.05 decrease and CAC per 100-Agatston-unit increase. Firth penalized Cox models were sensitivity analyses for diabetes and HRP because one group had no events; no multivariable survival model was fitted. Harrell's C-index summarized apparent univariable discrimination, without ROC-derived cutoffs. Stabilized inverse probability-of-censoring weights for follow-up completion included the four-level altitude–diabetes group, age, sex, body mass index, hypertension, smoking, LDL-C, HDL-C, triglycerides, HRP, LAD-PCAT, LAD CT-FFR, log2(CAC + 1), log(total plaque volume+1), and log(plaque burden + 1); weighted Cox models used matched-pair-robust variance.

The 13 altitude main-effect tests, 13 diabetes main-effect tests, and 13 altitude × diabetes interaction tests were treated as three separate multiplicity families and were adjusted separately using the Benjamini–Hochberg false discovery rate; raw *P* and q values are reported. Because the study was not sized *a priori* for interaction effects, nonsignificant interactions were considered inconclusive. All tests were two-sided with *P* < 0.05. Analyses used R 4.4.3 and Python 3.13.5 (statsmodels 0.14.4).

## Results

### Study population and baseline characteristics

The 248-participant source cohort yielded 174 matched participants in 87 pairs: 48 high-altitude diabetes (HD), 39 high-altitude non-diabetes (HN), 48 low-altitude diabetes (LD), and 39 low-altitude non-diabetes (LN). Among the 174 examinations, 25 (14.4%) were acquired with SOMATOM Definition Flash at 100 kVp and 149 (85.6%) with Revolution CT at 120 kVp; group-specific distributions are reported in Supplementary Table S1. Matching improved balance in age, sex, body mass index, and hypertension within both diabetes strata. Seven of eight post-match SMDs were below |0.10|; BMI in the diabetes stratum had |SMD| = 0.146. Pre- and post-match SMDs, propensity-score overlap, sample sizes, and characteristics of unmatched participants are reported in Supplementary Tables S2–S4 and Figures S1, S2. Compared with participants without diabetes, those with diabetes included higher proportions of men, hypertension, current smoking, and alcohol use and had higher body mass index, with additional differences in several metabolic measures (Table 1).

**Table 1:** Baseline clinical characteristics of the matched study population.

Variable	High-altitude DM	High-altitude Non-DM	Low-altitude DM	Low-altitude Non-DM	*P*-Alt	*P*-DM	*P*-Int
	(*n* = 48)	(*n* = 39)	(*n* = 48)	(*n* = 39)			
Demographics							
Age, years	63.5 (10.5)	62.4 (13.6)	63.0 (10.6)	61.4 (11.4)	.626	.491	.863
Male, *n* (%)	36 (75.0)	19 (48.7)	35 (72.9)	19 (48.7)	.863	.002	.863
BMI, kg/m²	26.4 (3.3)	25.0 (3.8)	26.8 (3.3)	25.3 (3.6)	.462	.015	.805
Clinical Characteristics							
Hypertension, *n* (%)	29 (60.4)	15 (38.5)	28 (58.3)	16 (41.0)	.967	.034	.702
Current smoking, *n* (%)	13 (27.1)	7 (17.9)	22 (45.8)	2 (5.1)	.557	<.001	.025
Alcohol use, *n* (%)	12 (25.0)	3 (7.7)	18 (37.5)	2 (5.1)	.886	<.001	.348
Metabolic Parameters							
FBG, mmol/L	8.3 (4.2)	5.1 (0.7)	7.5 (2.1)	5.1 (0.7)	.184	<.001	.194
HbA1c, %	7.6 (1.5)	5.7 (0.3)	7.1 (1.2)	5.6 (0.3)	.087	<.001	.223
TC, mmol/L	4.05 (1.21)	4.17 (1.01)	3.80 (0.89)	4.31 (0.93)	.741	.035	.239
TG, mmol/L	1.82 (1.24)	1.05 (0.35)	1.78 (0.66)	1.30 (0.54)	.299	<.001	.159
LDL-C, mmol/L	2.41 (1.00)	2.60 (0.90)	2.16 (0.75)	2.58 (0.75)	.329	.016	.411
HDL-C, mmol/L	1.01 (0.32)	1.17 (0.28)	1.04 (0.25)	1.31 (0.28)	.05	<.001	.215

Values are mean (SD) or *n* (%). *P*-Alt, *P*-DM, and *P*-Int are pair-cluster-robust tests. The smoking interaction is a baseline-covariate finding. BMI, body mass index; FBG, fasting blood glucose; HbA1c, glycated hemoglobin; TC, total cholesterol; TG, triglycerides; LDL-C, low-density lipoprotein cholesterol; HDL-C, high-density lipoprotein cholesterol.

Medication data were incomplete, with approximately 35% missingness across the available medication categories; observed use and missingness are summarized in Supplementary Table S5. Hemoglobin was available for all 174 participants, whereas oxygen saturation was available for 31 (17.8%) and was therefore retained for descriptive analysis only (Supplementary Table S6).

Among the 87 high-altitude participants in the matched cohort, 71 were in the 2,500–3,999 m subgroup and 16 were in the ≥4,000 m subgroup. Median residential altitude was 3,290 m (IQR, 2,650–3,650 m) and 4,225 m (IQR, 4,045–4,558 m), respectively. Apart from the expected difference in residential altitude, no statistically significant differences were observed in clinical or CCTA characteristics between the two subgroups (all *P* ≥ 0.137; Supplementary Table S7).

### Comprehensive CCTA imaging characteristics

In the primary covariate-adjusted matched models, diabetes remained associated with higher LAD-, LCX-, and RCA-PCAT, CAC, total plaque volume, plaque burden, and maximum stenosis and with lower LAD CT-FFR (raw *P* ≤ 0.011; *q* ≤ 0.018). Diabetes associations with HRP, LAP burden, LCX/RCA CT-FFR, and three-level CAD severity did not meet the FDR threshold (*q* ≥ 0.102) (Table 2; Supplementary Tables S8–S10).

**Table 2:** Comprehensive CCTA imaging characteristics by study group.

Variable	High-altitude DM	High-altitude Non-DM	Low-altitude DM	Low-altitude Non-DM	*P*-Alt	*P*-DM	*P*-Int
	(*n* = 48)	(*n* = 39)	(*n* = 48)	(*n* = 39)			
Pericoronary Adipose Tissue Attenuation (HU)							
LAD-PCAT	−75.81 ± 7.75	−76.44 ± 6.11	−73.71 ± 6.27	−80.87 ± 5.36	0.276	0.003	<0.001
LCX-PCAT	−70.96 ± 7.57	−73.44 ± 7.58	−70.69 ± 9.17	−77.08 ± 6.39	0.144	0.004	0.094
RCA-PCAT	−75.40 ± 8.35	−82.64 ± 9.42	−75.23 ± 8.27	−85.49 ± 8.21	0.309	<0.001	0.292
CT-derived Fractional Flow Reserve							
LAD CT-FFR	0.80 ± 0.09	0.85 ± 0.05	0.77 ± 0.11	0.80 ± 0.07	0.001	0.004	0.372
LCX CT-FFR	0.91 ± 0.04	0.92 ± 0.05	0.89 ± 0.08	0.91 ± 0.03	0.066	0.317	0.391
RCA CT-FFR	0.85 ± 0.10	0.87 ± 0.07	0.79 ± 0.13	0.84 ± 0.09	0.005	0.087	0.247
Coronary Artery Calcium							
Total CAC	40.33 [0.00–251.81]	0.20 [0.00–32.80]	84.43 [8.05–297.40]	8.04 [0.00–99.48]	0.011	0.011	0.796
Plaque Characteristics							
Total plaque volume, mm³	168.22 [33.60–564.77]	37.70 [0.00–77.78]	180.12 [61.25–365.00]	52.43 [21.97–183.40]	0.023	0.001	0.494
LAP burden, %	4.53 [1.14–10.23]	1.46 [0.00–5.12]	2.70 [1.61–6.60]	1.75 [0.07–5.89]	0.977	0.071	0.300
HRP present, *n* (%)	34 (70.8)	19 (48.7)	40 (83.3)	26 (66.7)	0.028	0.112	0.888
Maximum stenosis, %	65.75 ± 32.69	43.49 ± 38.37	74.11 ± 24.80	52.44 ± 36.31	0.058	0.002	0.924
Plaque burden, %	5.28 [2.50–9.37]	2.93 [0.00–5.79]	7.54 [3.60–11.18]	5.94 [2.43–9.89]	<0.001	0.011	0.348
Severity of CAD, *n* (%)					0.885	0.830	0.971
No CAD (0)	13 (27.1)	23 (59.0)	3 (6.2)	14 (35.9)			
Non-obstructive (1–2)	11 (22.9)	9 (23.1)	19 (39.6)	14 (35.9)			
Obstructive (≥3)	24 (50.0)	7 (17.9)	26 (54.2)	11 (28.2)			

Values are mean ± SD, median [IQR], or *n* (%). Primary models included altitude, diabetes, their interaction, age, sex, body mass index, and hypertension. Inferential analyses used log2(CAC + 1) for total CAC and log(x + 1) for total plaque volume, LAP burden, and plaque burden; descriptive values are presented on the original scale. Continuous outcomes were analyzed using matched-pair–clustered linear regression, HRP using matched-pair–clustered logistic regression, and CAD severity using multinomial logistic regression with matched-pair cluster bootstrap. *P*-Alt, *P*-DM, and *P*-Int denote the adjusted altitude, diabetes, and interaction tests, respectively. BH q values are reported in Supplementary Table S8 and complete estimates in Supplementary Table S10. CAD severity was classified according to CAD-RADS 2.0. CAC, coronary artery calcium; CT-FFR, CT-derived fractional flow reserve; HRP, high-risk plaque; HU, Hounsfield units; LAD, left anterior descending artery; LAP, low-attenuation plaque; LCX, left circumflex artery; PCAT, pericoronary adipose tissue attenuation; RCA, right coronary artery.

In the primary covariate-adjusted matched models, high-altitude residence was associated with higher LAD and RCA CT-FFR and lower CAC and plaque burden after FDR correction (raw *P* ≤ 0.011; *q* ≤ 0.037). Associations with total plaque volume and HRP prevalence did not meet the FDR threshold (both *q* = 0.060), and associations with PCAT were not statistically significant (*q* ≥ 0.208) (Table 2; Supplementary Tables S8–S10).

Among the 13 imaging interaction tests in the primary covariate-adjusted matched models, the altitude × diabetes interaction for LAD-PCAT was significant (interaction estimate −6.62 HU; 95% CI, −10.32 to −2.91; raw *P* < 0.001) and remained significant after Benjamini–Hochberg FDR correction (*q* = 0.008). The global multinomial interaction for CAD severity had raw *P* = 0.971 and did not meet the FDR threshold (*q* = 0.971); no other imaging interaction met the FDR threshold (Table 2; Supplementary Tables S8–S10). In the scanner-adjusted LAD-PCAT sensitivity model, the interaction estimate remained similar (−6.59 HU; 95% CI, −10.31 to −2.87; *P* < 0.001), and the scanner-type coefficient was −0.43 HU (95% CI, −3.66 to 2.81; *P* = 0.794). Corresponding pair-clustered effect estimates from the primary models are shown in Figure 3.

**Figure 3:**
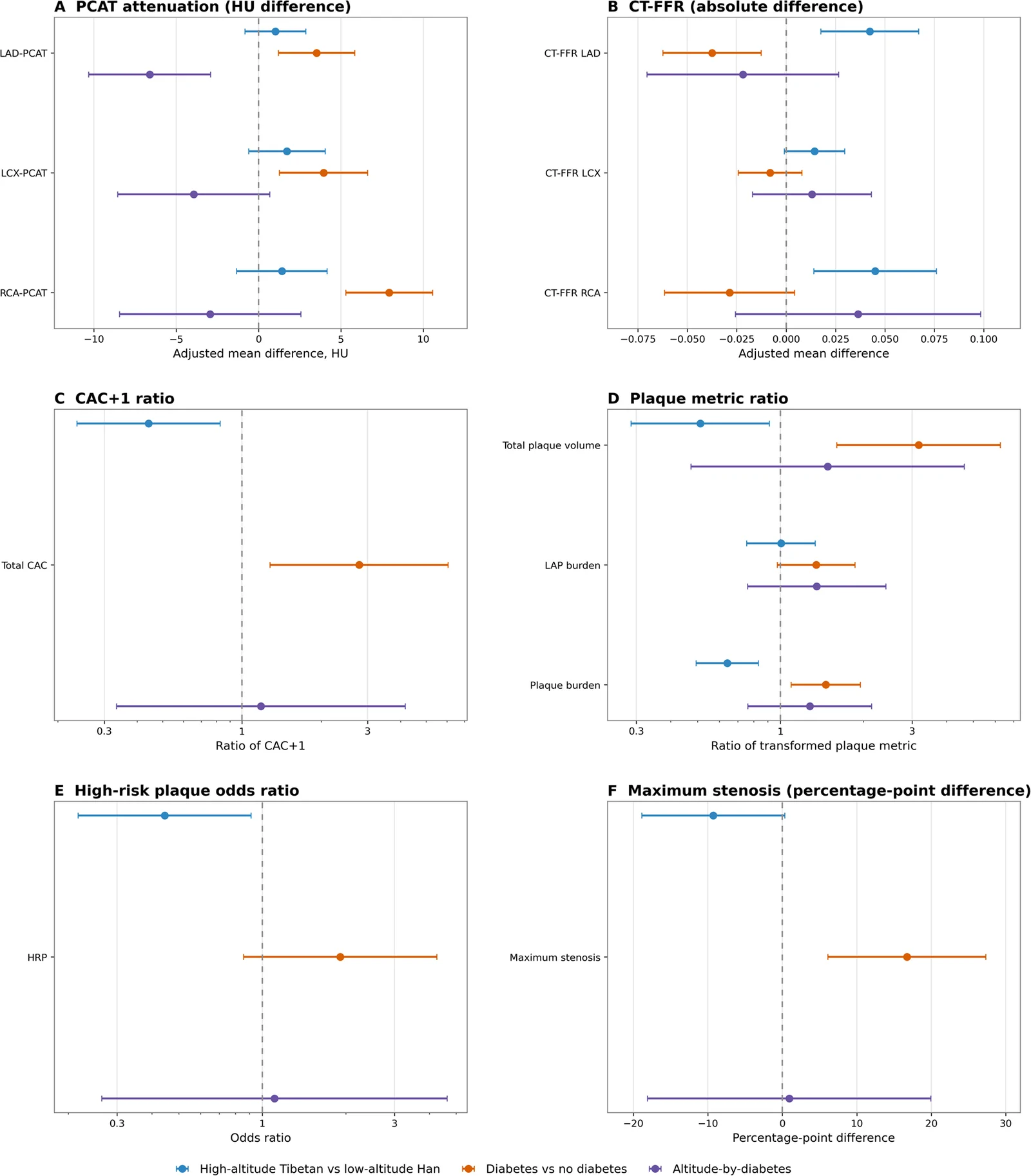
Primary covariate-adjusted matched-model effect estimates (points) with 95% confidence intervals (error bars) for high-altitude Tibetan versus low-altitude Han (blue), diabetes versus no diabetes (orange), and their interaction (purple). **(A)** PCAT attenuation difference in HU; **(B)** CT-FFR absolute difference; **(C)** ratio of CAC+1 based on the log2(CAC+1) model; **(D)** ratios for log(x + 1)-transformed plaque metrics; **(E)** odds ratio for HRP; and **(F)** maximum-stenosis percentage-point difference. Models were adjusted for age, sex, body mass index, and hypertension. Continuous outcomes used HC1 standard errors clustered by matched pair; HRP used pair-clustered logistic regression. Vertical dashed lines indicate the null value. CAC, coronary artery calcium; CI, confidence interval; CCTA, coronary computed tomography angiography; CT-FFR, CCTA-derived fractional flow reserve; HRP, high-risk plaque; HU, Hounsfield units; LAD, left anterior descending artery; LAP, low-attenuation plaque; LCX, left circumflex artery; PCAT, pericoronary adipose tissue attenuation; RCA, right coronary artery.

### Clinical outcomes and survival analysis

Of the 174 propensity score-matched patients, 119 (68.4%) completed clinical follow-up through electronic medical record review and structured telephone interviews. Follow-up completion rates were lower in high-altitude groups [HD: 30/48 [62.5%]; HN: 22/39 [56.4%]] than in low-altitude groups [LD: 37/48 [77.1%]; LN: 30/39 [76.9%]]. During a median follow-up of 28 months (IQR 16–38), MACE occurred in 20 of 119 participants (16.8%). The 20 events comprised all-cause death (*n* = 5), myocardial infarction (*n* = 2), ischemic stroke (*n* = 2), and coronary revascularization (*n* = 11) (Supplementary Table S11). Event rates differed markedly across groups, with the highest incidence in the LD group (29.7%) and no events in the LN group. Kaplan–Meier analysis demonstrated significantly different MACE-free survival among the four study groups (log-rank *P* = 0.001), primarily driven by diabetes status (log-rank *P* < 0.001; Figure 4). Baseline characteristics of participants with and without follow-up are compared in Supplementary Table S12; participants who completed follow-up included lower proportions of high-altitude residents (43.7% vs. 63.6%, *P* = 0.014) and men (57.1% vs. 74.5%, *P* = 0.027), and had higher HDL-C levels (1.15 ± 0.31 vs. 1.05 ± 0.29 mmol/L, *P* = 0.046), indicating potential attrition bias.

**Figure 4:**
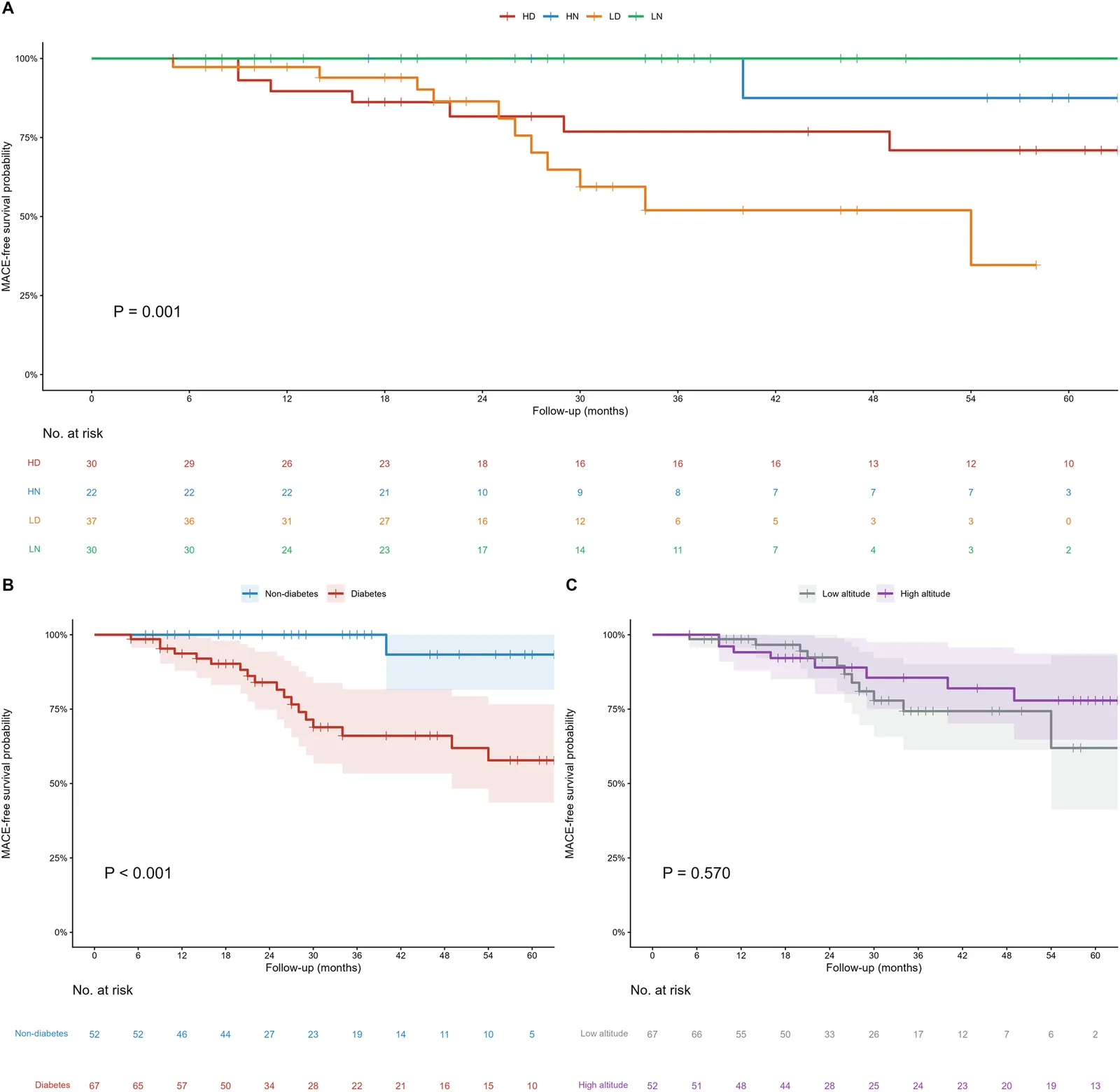
Kaplan–Meier estimates of MACE-free survival. (A) HD, HN, LD, and LN groups; (B) diabetes versus no diabetes; and (C) high- versus low-altitude residence. The *y*-axis in each panel is labeled MACE-free survival probability. Tick marks indicate censoring; shaded bands in panels B and C are 95% confidence intervals; numbers at risk are shown below each panel. *P* values are from log-rank tests. Curves are descriptive because follow-up was available for 119 participants and only 20 MACE occurred. CI, confidence interval; HD, high-altitude diabetes; HN, high-altitude non-diabetes; LD, low-altitude diabetes; LN, low-altitude non-diabetes; MACE, major adverse cardiovascular events.

In exploratory pair-robust univariable Cox analyses (Table 3), diabetes (HR = 14.95; 95% CI, 2.21–101.01; *P* = 0.006) and HRP (HR = 14.94; 95% CI, 1.99–112.32; *P* = 0.009) were associated with MACE, although both estimates were imprecise. Altitude was not statistically associated with MACE (HR = 0.77; 95% CI, 0.32–1.84; *P* = 0.557). Several CCTA measures, including PCAT attenuation, CT-FFR, CAC, plaque burden, and stenosis measures, also showed univariable associations with MACE (Table 3). Firth-penalized Cox analyses for diabetes and HRP yielded directionally consistent but imprecise estimates (Supplementary Table S13). IPCW sensitivity analyses generally preserved the direction of associations, although the association between LAD-PCAT and MACE was attenuated (Supplementary Table S14). Because all survival models were univariable, these findings do not establish independent prognostic associations.

**Table 3:** Exploratory pair-robust univariable Cox associations with MACE.

Variable	HR	95% CI	*P*
Clinical variables			
Altitude (high vs. low)	0.771	0.324–1.836	0.557
Diabetes (yes vs. no)	14.948	2.212–101.009	0.006
Age (per 10 years)	1.485	1.011–2.181	0.044
Sex (male vs. female)	1.406	0.601–3.288	0.432
Hypertension	0.893	0.344–2.323	0.817
Smoking	3.166	1.337–7.497	0.009
Plaque characteristics			
HRP (present)	14.938	1.987–112.324	0.009
Pericoronary adipose tissue attenuation			
LAD-PCAT (per 5 HU increase)	1.54	1.136–2.087	0.005
LCX-PCAT (per 5 HU increase)	1.482	1.155–1.901	0.002
RCA-PCAT (per 5 HU increase)	1.48	1.157–1.894	0.002
CT-derived fractional flow reserve			
CT-FFR LAD (per 0.05 decrease)	1.431	1.241–1.649	<0.001
CT-FFR LCX (per 0.05 decrease)	1.371	1.208–1.556	<0.001
CT-FFR RCA (per 0.05 decrease)	1.218	1.075–1.380	0.002
Coronary artery calcium			
CAC (per 100 Agatston units)	1.165	1.095–1.240	<0.001
Plaque characteristics			
Total plaque volume (per 100 mm^3^)	1.131	1.058–1.209	<0.001
Plaque burden (per 5 percentage points)	1.67	1.264–2.206	<0.001
Maximum stenosis (per 10 percentage points)	1.394	1.207–1.609	<0.001
Severity of coronary artery disease			
CAD severity (per category)	6.074	2.647–13.938	<0.001

*N* = 119; events = 20. Each row is a separate univariable Cox model with matched-pair robust variance; estimates are not mutually adjusted or independently validated. The proportional-hazards assumption was formally assessed for all 18 models using scaled Schoenfeld residuals (cox.zph with the Kaplan–Meier time transformation); global *P* values ranged from 0.227 to 0.959, with no *P* < 0.05 (Supplementary Table S15B). CAC, coronary artery calcium; CAD-RADS, Coronary Artery Disease–Reporting and Data System; CI, confidence interval; CT-FFR, CT-derived fractional flow reserve; HR, hazard ratio; HRP, high-risk plaque; HU, Hounsfield units; LAD, left anterior descending artery; LAP, low-attenuation plaque; LCX, left circumflex artery; MACE, major adverse cardiovascular events; PCAT, pericoronary adipose tissue attenuation; RCA, right coronary artery.

Apparent univariable discrimination for the censored outcome was summarized using Harrell's C-index. Total plaque volume had the highest apparent C-index (0.811; 95% CI 0.720–0.901), but this in-sample estimate was exploratory and no clinical threshold was derived (Supplementary Figure S3, Table S15A).

## Discussion

This study comprehensively evaluated CCTA-derived imaging biomarkers spanning plaque characteristics, pericoronary inflammation, coronary calcification, and hemodynamic function in patients with and without diabetes who resided at high or low altitude. We used a matched 2 × 2 design based on altitude and diabetes status, with retrospective baseline assessment and prospective outcome ascertainment. This design allowed us to characterize the associations of altitude and diabetes with coronary imaging phenotypes and to explore their relationships with cardiovascular outcomes.

In the primary covariate-adjusted imaging analysis, diabetes was associated with a broadly adverse CCTA phenotype. After adjustment for age, sex, body mass index, and hypertension, associations with higher PCAT attenuation, CAC, total plaque volume, plaque burden, and maximum stenosis, as well as lower LAD CT-FFR, remained significant after FDR correction. In contrast, associations with HRP, LAP burden, and LCX and RCA CT-FFR did not meet the FDR threshold. These findings are consistent with previous evidence linking diabetes to greater coronary atherosclerotic burden and cardiovascular risk (7, 12, 29). Compared with the low-altitude Han group, the high-altitude Tibetan group showed a distinct imaging profile after adjustment, characterized by higher LAD and RCA CT-FFR and lower CAC and plaque burden after FDR correction. In the exploratory survival analysis, diabetes showed one of the largest univariable associations with MACE, although the wide confidence interval and absence of multivariable modeling preclude conclusions regarding independent prognostic importance. Because all CCTA examinations were performed at low-altitude West China Hospital, the observed CT-FFR differences reflect CCTA-derived physiology under the examination conditions rather than physiology measured during hypobaric exposure. In addition, altitude was completely aligned with ethnicity, and the high-altitude group consisted of Tibetan patients who were able to travel to a tertiary center for clinically indicated CCTA. These findings should therefore be interpreted cautiously and confirmed in larger, ethnically comparable multicenter cohorts.

Evidence regarding the relationship between long-term high-altitude residence and cardiovascular outcomes remains limited and may depend on the clinical setting. Lei et al. studied 3,020 patients undergoing cardiac surgery and identified 377 propensity-matched altitude pairs, defining high altitude as ≥1,500 m and low altitude as <1,500 m. They reported a lower incidence of perioperative MACE among high-altitude residents than among low-altitude residents (6.6% vs. 11.9%, *P* = 0.017) (30). Their composite outcome primarily reflected intraoperative and postoperative ischemia-reperfusion complications, including myocardial infarction requiring intervention, cardiac arrest, and low-output or acute heart failure requiring mechanical circulatory support. Our study addressed a different clinical context. We compared residents at ≥2,500 m with those at <500 m in a clinically indicated CCTA cohort of 174 matched participants, of whom 119 had follow-up and 20 experienced MACE during a median follow-up of 28 months. Our composite outcome included all-cause death, myocardial infarction, revascularization for refractory angina, and ischemic stroke. Thus, the two studies differed substantially in population, altitude definition, pathophysiologic setting, outcome definition, and follow-up window. The lower perioperative event rate reported by Lei et al. and the nonsignificant association between altitude and MACE in our cohort should therefore not be considered contradictory. Given the limited number of events and differential follow-up in our cohort, we cannot confirm or exclude an association between high-altitude residence and longer-term cardiovascular outcomes.

Among the 13 imaging interaction tests, LAD-PCAT was the only measure that remained significant after multiplicity correction in the primary covariate-adjusted model. This finding indicates that the association between diabetes and LAD-PCAT attenuation differed between the high-altitude Tibetan and low-altitude Han groups. Descriptively, LAD-PCAT attenuation was higher in high-altitude Tibetan than in low-altitude Han participants without diabetes, whereas the difference between groups was smaller and in the opposite direction among participants with diabetes. Although all three vessel-specific PCAT measures were associated with MACE in exploratory univariable analyses, the limited number of events precluded assessment of their independent prognostic value or whether these associations differed between groups. Prior meta-analytic evidence supports PCAT attenuation as a prognostic marker in broader populations (31). The interaction estimate was similar after scanner-type adjustment in a sensitivity model. Our findings therefore raise the possibility that diabetes-related differences in LAD-PCAT attenuation may differ between the high-altitude Tibetan and low-altitude Han groups, but this observation requires validation in larger independent cohorts.

HRP showed a strong but imprecise univariable association with MACE, although the limited number of events and absence of multivariable survival modeling precluded assessment of its independent prognostic value. Previous analyses from PROMISE and SCOT-HEART have also shown that the prognostic association of HRP may be attenuated after accounting for other clinical and imaging characteristics (32, 33). In our adjusted imaging analysis, the association between diabetes and HRP was attenuated, whereas HRP prevalence differed between the high-altitude Tibetan and low-altitude Han groups. This finding further supports the presence of distinct coronary imaging phenotypes between these groups. However, its underlying mechanisms and clinical implications remain uncertain because ethnicity, referral selection, treatment, and other unmeasured factors may have contributed to the observed association.

## Limitations

This study has several limitations. First, follow-up was unavailable for 55 of 174 matched participants, and completion rates differed by altitude. Baseline differences between participants with and without follow-up, together with attenuation of the LAD-PCAT association with MACE in the IPCW analysis, indicate potential attrition bias. Second, only 20 MACE occurred, one study group had no events, and several effect estimates had wide confidence intervals. Accordingly, all survival analyses were exploratory and univariable, and no conclusions regarding independent prognostic value can be drawn. Because revascularization was included in MACE, baseline CCTA findings may have influenced subsequent management and event classification. The matched cohort was also not sized *a priori* to detect altitude × diabetes interactions, so modest interactions may have been missed. Nonsignificant interaction tests should therefore be considered inconclusive rather than evidence of no interaction. Third, PSM was based on age, sex, BMI, and hypertension, with residual imbalance in BMI in the diabetes stratum (SMD=0.146). The covariate-adjusted models included the same four variables but did not account for lipid profiles, HbA1c, fasting glucose, medication use, or diabetes duration. Medication data were approximately 35% incomplete, diabetes duration was available for 67 participants, and oxygen saturation for 31. Residual confounding related to metabolic control, treatment, and physiologic status therefore remains likely. Fourth, altitude and ethnicity were completely aligned. All high-altitude participants were Tibetan, whereas all low-altitude participants were Han. The observed altitude-associated differences may therefore reflect ancestry and long-term genetic adaptation, including EPAS1-related pathways (34), as well as referral selection, socioeconomic conditions, healthcare access, urban versus rural residence, ability to travel, and health awareness. Altitude-specific causal effects cannot be separated from these factors in the present cohort. Fifth, all CCTA examinations were performed at a low-altitude center. CT-FFR therefore reflects physiology under the examination conditions rather than direct measurement during hypobaric exposure. Sixth, severe chronic respiratory disease, including chronic obstructive pulmonary disease, HIV/AIDS, tuberculosis, and other major systemic conditions were not prespecified exclusion criteria and were not systematically excluded. Exact residence duration beyond the 5-year threshold was unavailable, and only 16 participants resided at ≥4,000 m, limiting assessment of exposure duration and very-high-altitude effects. Finally, the single-center design, use of two CT scanners, reliance on a single uAI analysis platform, and median 28-month follow-up limit generalizability and long-term inference.

## Conclusions

In this clinically indicated CCTA cohort, diabetes was associated with a more adverse coronary imaging phenotype, including higher PCAT attenuation, CAC, total plaque volume, plaque burden, and maximum stenosis, together with lower LAD CT-FFR. Compared with the low-altitude Han group, the high-altitude Tibetan group showed a distinct imaging profile characterized by higher LAD and RCA CT-FFR and lower CAC and plaque burden. The association between diabetes and LAD-PCAT attenuation differed between these groups. Given the limited number of events and differential follow-up, the prognostic implications of these findings remain uncertain. Larger multicenter longitudinal studies with ethnically comparable populations and comprehensive assessment of metabolic control and treatment are needed to validate these associations and clarify their clinical relevance.

## Data Availability

The datasets used and analyzed during the current study are available from the corresponding author on reasonable request.
