# Supplementary material for: Comprehensive coronary computed tomography angiography assessment in high-altitude diabetic populations: integrating plaque characteristics, pericoronary inflammation, and coronary function
Source: Front Cardiovasc Med. 2026 Oct 9;13:1886360. doi: 10.3389/fcvm.2026.1886360 (PMC13690624; doi:10.3389/fcvm.2026.1886360)
Supplement: Supplementary file 1 [file Datasheet1.docx]

**SUPPLEMENTARY MATERIAL**

Supplementary Table S1. Scanner type and tube voltage distribution across the four study groups

| **Scanner type / tube voltage** | **Overall** | **High-altitude diabetes** | **High-altitude no diabetes** | **Low-altitude diabetes** | **Low-altitude no diabetes** |
| --- | --- | --- | --- | --- | --- |
| SOMATOM Definition Flash, 100 kVp | 25/174 (14.4%) | 15/48 (31.3%) | 10/39 (25.6%) | 0/48 (0.0%) | 0/39 (0.0%) |
| Revolution CT, 120 kVp | 149/174 (85.6%) | 33/48 (68.8%) | 29/39 (74.4%) | 48/48 (100.0%) | 39/39 (100.0%) |

Values are n/N (%).

Supplementary Table S2. Propensity-score balance before and after matching

| **Stratum** | **Phase** | **Variable** | **High altitude** | **Low altitude** | **SMD** |
| --- | --- | --- | --- | --- | --- |
| T2DM | Pre-matching | Age | 62.10 ± 11.26 | 64.93 ± 11.24 | -0.254 |
| T2DM | Pre-matching | Sex (male) | 48/62 (77.4%) | 39/59 (66.1%) | 0.253 |
| T2DM | Pre-matching | BMI | 27.09 ± 3.66 | 25.98 ± 3.59 | 0.310 |
| T2DM | Pre-matching | Hypertension | 31/62 (50.0%) | 38/59 (64.4%) | -0.294 |
| T2DM | Post-matching | Age | 63.52 ± 10.46 | 63.02 ± 10.58 | 0.048 |
| T2DM | Post-matching | Sex (male) | 36/48 (75.0%) | 35/48 (72.9%) | 0.047 |
| T2DM | Post-matching | BMI | 26.35 ± 3.28 | 26.83 ± 3.28 | -0.146 |
| T2DM | Post-matching | Hypertension | 29/48 (60.4%) | 28/48 (58.3%) | 0.042 |
| Non-T2DM | Pre-matching | Age | 59.68 ± 12.52 | 65.39 ± 11.51 | -0.479 |
| Non-T2DM | Pre-matching | Sex (male) | 30/66 (45.5%) | 29/61 (47.5%) | -0.042 |
| Non-T2DM | Pre-matching | BMI | 26.35 ± 3.99 | 24.65 ± 3.61 | 0.450 |
| Non-T2DM | Pre-matching | Hypertension | 17/66 (25.8%) | 31/61 (50.8%) | -0.534 |
| Non-T2DM | Post-matching | Age | 62.44 ± 13.63 | 61.38 ± 11.44 | 0.085 |
| Non-T2DM | Post-matching | Sex (male) | 19/39 (48.7%) | 19/39 (48.7%) | 0.000 |
| Non-T2DM | Post-matching | BMI | 25.02 ± 3.76 | 25.26 ± 3.59 | -0.065 |
| Non-T2DM | Post-matching | Hypertension | 15/39 (38.5%) | 16/39 (41.0%) | -0.052 |

Matching was performed separately within diabetes strata using age, sex, BMI, and hypertension. SMD is high altitude minus low altitude; |SMD|<0.10 was the reference criterion. Seven of eight post-match SMDs met this criterion; T2DM-stratum BMI had |SMD|=0.146. BMI, body mass index; SMD, standardized mean difference; T2DM, type 2 diabetes mellitus.

Supplementary Table S3. Propensity-score overlap summary

| **Diabetes stratum** | **Altitude** | **Match status** | **N** | **Median [IQR]** | **Range** |
| --- | --- | --- | --- | --- | --- |
| T2DM | High | Unmatched | 14 | 0.665 [0.648–0.705] | 0.542–0.786 |
| T2DM | High | Matched | 48 | 0.514 [0.425–0.579] | 0.276–0.754 |
| T2DM | Low | Unmatched | 11 | 0.314 [0.267–0.361] | 0.224–0.375 |
| T2DM | Low | Matched | 48 | 0.531 [0.432–0.589] | 0.270–0.764 |
| Non-T2DM | High | Unmatched | 27 | 0.725 [0.693–0.766] | 0.638–0.956 |
| Non-T2DM | High | Matched | 39 | 0.530 [0.421–0.620] | 0.106–0.837 |
| Non-T2DM | Low | Unmatched | 22 | 0.295 [0.250–0.327] | 0.092–0.536 |
| Non-T2DM | Low | Matched | 39 | 0.533 [0.425–0.642] | 0.096–0.829 |

Propensity scores were estimated separately within diabetes strata using age, sex, BMI, and hypertension. Values summarize the source-cohort distribution according to altitude and subsequent match status. Individual identifiers are not shown.

Supplementary Table S4. Characteristics of matched versus unmatched source participants

| **Variable** | **Matched (n=174)** | **Unmatched (n=74)** | **SMD** |
| --- | --- | --- | --- |
| Age, years | 62.66 (11.41); N=174 | 63.59 (12.80); N=74 | -0.077 |
| BMI, kg/m² | 25.94 (3.52); N=174 | 26.23 (4.43); N=74 | -0.073 |
| FBG, mmol/L | 6.65 (2.85); N=174 | 6.67 (3.29); N=74 | -0.005 |
| HbA1c, % | 6.98 (1.43); N=87 | 7.12 (2.10); N=29 | -0.079 |
| Triglycerides, mmol/L | 1.52 (0.85); N=174 | 1.43 (0.78); N=74 | 0.114 |
| Total cholesterol, mmol/L | 4.07 (1.03); N=174 | 4.43 (1.12); N=74 | -0.336 |
| HDL-C, mmol/L | 1.12 (0.30); N=174 | 1.21 (0.35); N=74 | -0.274 |
| LDL-C, mmol/L | 2.42 (0.87); N=174 | 2.74 (0.90); N=74 | -0.366 |
| High altitude | 87/174 (50.0%) | 41/74 (55.4%) | -0.108 |
| Diabetes | 96/174 (55.2%) | 25/74 (33.8%) | 0.441 |
| Male | 109/174 (62.6%) | 37/74 (50.0%) | 0.257 |
| Hypertension | 88/174 (50.6%) | 29/74 (39.2%) | 0.230 |
| Current smoking | 44/174 (25.3%) | 18/74 (24.3%) | 0.022 |

Values are mean (SD); available N or n/N (%). SMD compares matched with unmatched source participants and is descriptive.

Supplementary Table S5. Observed medication use and missingness

| **Medication data** | **Overall** | **Diabetes** | **No diabetes** | **High altitude** | **Low altitude** |
| --- | --- | --- | --- | --- | --- |
| ***Observed medication use, n/N (%)*** |  |  |  |  |  |
| Antiplatelet | 25/114 (21.9) | 20/62 (32.3) | 5/52 (9.6) | 14/52 (26.9) | 11/62 (17.7) |
| Statin | 22/113 (19.5) | 20/62 (32.3) | 2/51 (3.9) | 13/51 (25.5) | 9/62 (14.5) |
| ACEI/ARB | 3/114 (2.6) | 2/62 (3.2) | 1/52 (1.9) | 1/52 (1.9) | 2/62 (3.2) |
| Beta-blocker | 0/114 (0.0) | 0/62 (0.0) | 0/52 (0.0) | 0/52 (0.0) | 0/62 (0.0) |
| Glucose-lowering medication | 25/114 (21.9) | 22/62 (35.5) | 3/52 (5.8) | 11/52 (21.2) | 14/62 (22.6) |
| Insulin | 1/113 (0.9) | 1/61 (1.6) | 0/52 (0.0) | 1/51 (2.0) | 0/62 (0.0) |
| ***Missing, n (%)*** |  |  |  |  |  |
| Antiplatelet | 60 (34.5) | 34 (35.4) | 26 (33.3) | 35 (40.2) | 25 (28.7) |
| Statin | 61 (35.1) | 34 (35.4) | 27 (34.6) | 36 (41.4) | 25 (28.7) |
| ACEI/ARB | 60 (34.5) | 34 (35.4) | 26 (33.3) | 35 (40.2) | 25 (28.7) |
| Beta-blocker | 60 (34.5) | 34 (35.4) | 26 (33.3) | 35 (40.2) | 25 (28.7) |
| Glucose-lowering medication | 60 (34.5) | 34 (35.4) | 26 (33.3) | 35 (40.2) | 25 (28.7) |
| Insulin | 61 (35.1) | 35 (36.5) | 26 (33.3) | 36 (41.4) | 25 (28.7) |

Observed use is n/N among nonmissing records. Missing values were not interpreted as nonuse, were not imputed, and were not included in primary adjustment. Separate SGLT2-inhibitor and GLP-1 receptor agonist fields were unavailable.

Supplementary Table S6. Physiologic and diabetes-duration availability and descriptive summaries

| **Variable** | **Overall** | **High altitude** | **Low altitude** | **Available N** | **Missing, n (%)** |
| --- | --- | --- | --- | --- | --- |
| Hemoglobin, g/L | 139.99 ± 21.53 | 142.70 ± 25.25 | 137.29 ± 16.73 | 174 | 0 (0.0) |
| Oxygen saturation, % | 96.57 ± 2.69 (n=31) | 96.38 ± 2.84 (n=25) | 97.38 ± 1.90 (n=6) | 31 | 143 (82.2) |
| Reported diabetes duration, years | 9.00 [4.00–13.50] (n=67) | 10.00 [5.00–10.00] (n=22) | 9.00 [3.00–17.00] (n=45) | 67 | 107 (61.5) |

Values are mean ± SD for hemoglobin and oxygen saturation and median [IQR] for reported diabetes duration. All summaries are descriptive. Hemoglobin was treated as a physiologic descriptor; oxygen saturation and diabetes duration were not included in adjustment models because of limited availability.

Supplementary Table S7. Clinical and CCTA characteristics of high-altitude participants according to residential altitude

| **Variable** | **2,500–3,999 m (n=71)** | **≥4,000 m (n=16)** | **P value** |
| --- | --- | --- | --- |
| ***Residential altitude and clinical characteristics*** | | | |
| Residential altitude, m | 3290 [2650–3650] | 4225 [4045–4558] | <0.001 |
| Age, years | 62.8 ± 12.0 | 64.1 ± 12.0 | 0.708 |
| Male, n (%) | 44/71 (62.0) | 11/16 (68.8) | 0.776 |
| BMI, kg/m² | 25.8 ± 3.5 | 25.5 ± 3.9 | 0.802 |
| Diabetes, n (%) | 40/71 (56.3) | 8/16 (50.0) | 0.782 |
| Hypertension, n (%) | 34/71 (47.9) | 10/16 (62.5) | 0.408 |
| Current smoking, n (%) | 16/71 (22.5) | 4/16 (25.0) | 1.000 |
| ***CCTA characteristics*** | | | |
| LAD-PCAT, HU | -76.35 ± 6.90 | -74.94 ± 7.68 | 0.505 |
| LCX-PCAT, HU | -73.00 [-76.00–-68.00] | -71.00 [-78.00–-63.00] | 0.780 |
| RCA-PCAT, HU | -78.82 ± 8.56 | -77.88 ± 13.25 | 0.789 |
| LAD CT-FFR | 0.84 [0.80–0.87] | 0.84 [0.82–0.87] | 0.750 |
| LCX CT-FFR | 0.93 [0.90–0.95] | 0.93 [0.88–0.93] | 0.460 |
| RCA CT-FFR | 0.89 [0.83–0.91] | 0.88 [0.79–0.91] | 0.294 |
| Total CAC | 3.18 [0.00–139.59] | 3.98 [0.00–159.65] | 0.713 |
| Total plaque volume, mm³ | 133.47 [52.32–457.19] | 249.72 [75.92–1034.40] | 0.264 |
| LAP burden, % | 3.26 [0.16–8.09] | 1.57 [0.00–3.78] | 0.137 |
| HRP present, n (%) | 44/71 (62.0) | 9/16 (56.2) | 0.779 |
| Maximum stenosis, % | 65.41 [34.18–84.68] | 51.25 [0.00–77.70] | 0.248 |
| Plaque burden, % | 4.47 [1.09–8.05] | 3.04 [0.00–6.87] | 0.450 |
| Obstructive CAD, n (%) | 26/71 (36.6) | 5/16 (31.2) | 0.779 |

Values are mean ± SD, median [IQR], or n/N (%). Approximately normally distributed continuous variables were compared using Welch's t test and other continuous variables using the Mann–Whitney U test; categorical variables were compared using Fisher's exact test. P values are two-sided, unadjusted, and should be interpreted descriptively because this was an exploratory subgroup comparison. All high-altitude participants met the prespecified ≥5-year residence criterion; exact individual residence duration beyond this threshold was unavailable.

Supplementary Table S8. Multiplicity-adjusted primary imaging analyses

| **Outcome** | **P-altitude** | **BH q-altitude** | **P-diabetes** | **BH q-diabetes** | **P-interaction** | **BH q-interaction** |
| --- | --- | --- | --- | --- | --- | --- |
| LAD-PCAT | 0.276 | 0.359 | 0.003 | 0.009 | <0.001 | 0.008 |
| LCX-PCAT | 0.144 | 0.208 | 0.004 | 0.009 | 0.094 | 0.612 |
| RCA-PCAT | 0.309 | 0.366 | <0.001 | <0.001 | 0.292 | 0.635 |
| CT-FFR LAD | 0.001 | 0.007 | 0.004 | 0.009 | 0.372 | 0.635 |
| CT-FFR LCX | 0.066 | 0.107 | 0.317 | 0.343 | 0.391 | 0.635 |
| CT-FFR RCA | 0.005 | 0.021 | 0.087 | 0.113 | 0.247 | 0.635 |
| Total CAC | 0.011 | 0.037 | 0.011 | 0.018 | 0.796 | 0.971 |
| Total plaque volume | 0.023 | 0.060 | 0.001 | 0.008 | 0.494 | 0.714 |
| LAP burden | 0.977 | 0.977 | 0.071 | 0.102 | 0.300 | 0.635 |
| HRP | 0.028 | 0.060 | 0.112 | 0.132 | 0.888 | 0.971 |
| Maximum stenosis | 0.058 | 0.107 | 0.002 | 0.009 | 0.924 | 0.971 |
| Plaque burden | <0.001 | 0.007 | 0.011 | 0.018 | 0.348 | 0.635 |
| CAD severity | 0.885 | 0.959 | 0.830 | 0.830 | 0.971 | 0.971 |

Primary models were adjusted for age, sex, body mass index, and hypertension. The Benjamini–Hochberg procedure was applied separately to the 13 altitude main-effect tests, 13 diabetes main-effect tests, and 13 altitude × diabetes interaction tests. CAD severity used adjusted global multinomial tests with 500 matched-pair bootstrap replicates. Raw P and BH q values are shown; q<0.05 was considered statistically significant.

Supplementary Table S9. Categorical imaging outcome models

| **Outcome** | **Model** | **Term** | **Effect estimate** | **95% CI** | **df** | **P** | **Bootstrap replicates** |
| --- | --- | --- | --- | --- | --- | --- | --- |
| HRP | Pair-clustered binary logistic | Altitude main association | OR 0.48 | 0.246–0.938 | 1 | 0.032 |  |
| HRP | Pair-clustered binary logistic | Diabetes main association | OR 2.528 | 1.242–5.147 | 1 | 0.011 |  |
| HRP | Pair-clustered binary logistic | Altitude × diabetes interaction | OR 1.023 | 0.268–3.902 | 1 | 0.974 |  |
| CAD severity | Multinomial logit with matched-pair cluster bootstrap | Altitude main association | Wald χ² 2.134 |  | 2 | 0.344 | 500 |
| CAD severity | Multinomial logit with matched-pair cluster bootstrap | Diabetes main association | Wald χ² 1.661 |  | 2 | 0.436 | 500 |
| CAD severity | Multinomial logit with matched-pair cluster bootstrap | Altitude × diabetes interaction | Wald χ² 9.384 |  | 2 | 0.009 | 500 |

HRP estimates are odds ratios from pair-clustered binary logistic regression. CAD-severity entries are global Wald tests from multinomial logistic regression with covariance estimated from 500 successful matched-pair cluster bootstrap replicates.

Supplementary Table S10. Primary covariate-adjusted imaging models in the matched cohort

| **Outcome** | Term | Adjusted estimate (95% CI) or global test | **P** |
| --- | --- | --- | --- |
| LAD-PCAT — Difference, HU | Altitude (high vs low) | 1.02 (-0.83, 2.87) | 0.276 |
| LAD-PCAT — Difference, HU | Diabetes (yes vs no) | 3.52 (1.21, 5.83) | 0.003 |
| LAD-PCAT — Difference, HU | Altitude × diabetes | -6.62 (-10.32, -2.91) | <0.001 |
| LCX-PCAT — Difference, HU | Altitude (high vs low) | 1.72 (-0.60, 4.04) | 0.144 |
| LCX-PCAT — Difference, HU | Diabetes (yes vs no) | 3.94 (1.27, 6.61) | 0.004 |
| LCX-PCAT — Difference, HU | Altitude × diabetes | -3.93 (-8.55, 0.69) | 0.094 |
| RCA-PCAT — Difference, HU | Altitude (high vs low) | 1.41 (-1.33, 4.16) | 0.309 |
| RCA-PCAT — Difference, HU | Diabetes (yes vs no) | 7.93 (5.29, 10.57) | <0.001 |
| RCA-PCAT — Difference, HU | Altitude × diabetes | -2.94 (-8.44, 2.57) | 0.292 |
| CT-FFR LAD — Difference | Altitude (high vs low) | 0.042 (0.018, 0.067) | 0.001 |
| CT-FFR LAD — Difference | Diabetes (yes vs no) | -0.037 (-0.062, -0.013) | 0.004 |
| CT-FFR LAD — Difference | Altitude × diabetes | -0.022 (-0.070, 0.027) | 0.372 |
| CT-FFR LCX — Difference | Altitude (high vs low) | 0.014 (-0.001, 0.030) | 0.066 |
| CT-FFR LCX — Difference | Diabetes (yes vs no) | -0.008 (-0.024, 0.008) | 0.317 |
| CT-FFR LCX — Difference | Altitude × diabetes | 0.013 (-0.017, 0.043) | 0.391 |
| CT-FFR RCA — Difference | Altitude (high vs low) | 0.045 (0.014, 0.076) | 0.005 |
| CT-FFR RCA — Difference | Diabetes (yes vs no) | -0.029 (-0.062, 0.004) | 0.087 |
| CT-FFR RCA — Difference | Altitude × diabetes | 0.036 (-0.026, 0.098) | 0.247 |
| Total CAC — Ratio of CAC+1 | Altitude (high vs low) | 0.44 (0.24, 0.83) | 0.011 |
| Total CAC — Ratio of CAC+1 | Diabetes (yes vs no) | 2.78 (1.28, 6.07) | 0.011 |
| Total CAC — Ratio of CAC+1 | Altitude × diabetes | 1.18 (0.33, 4.17) | 0.796 |
| Total plaque volume — Ratio of volume+1 | Altitude (high vs low) | 0.51 (0.29, 0.91) | 0.023 |
| Total plaque volume — Ratio of volume+1 | Diabetes (yes vs no) | 3.17 (1.60, 6.27) | 0.001 |
| Total plaque volume — Ratio of volume+1 | Altitude × diabetes | 1.48 (0.47, 4.64) | 0.494 |
| LAP burden — Ratio of burden+1 | Altitude (high vs low) | 1.00 (0.75, 1.34) | 0.977 |
| LAP burden — Ratio of burden+1 | Diabetes (yes vs no) | 1.35 (0.97, 1.86) | 0.071 |
| LAP burden — Ratio of burden+1 | Altitude × diabetes | 1.35 (0.76, 2.41) | 0.300 |
| HRP — Odds ratio | Altitude (high vs low) | 0.45 (0.22, 0.91) | 0.028 |
| HRP — Odds ratio | Diabetes (yes vs no) | 1.91 (0.86, 4.26) | 0.112 |
| HRP — Odds ratio | Altitude × diabetes | 1.11 (0.26, 4.64) | 0.888 |
| Maximum stenosis — Difference, percentage points | Altitude (high vs low) | -9.27 (-18.86, 0.32) | 0.058 |
| Maximum stenosis — Difference, percentage points | Diabetes (yes vs no) | 16.72 (6.12, 27.31) | 0.002 |
| Maximum stenosis — Difference, percentage points | Altitude × diabetes | 0.92 (-18.09, 19.93) | 0.924 |
| Plaque burden — Ratio of burden+1 | Altitude (high vs low) | 0.64 (0.49, 0.83) | <0.001 |
| Plaque burden — Ratio of burden+1 | Diabetes (yes vs no) | 1.46 (1.09, 1.95) | 0.011 |
| Plaque burden — Ratio of burden+1 | Altitude × diabetes | 1.28 (0.76, 2.14) | 0.348 |
| CAD severity | Altitude (high vs low) | Global Wald χ²=0.244 (df=2) | 0.885 |
| CAD severity | Diabetes (yes vs no) | Global Wald χ²=0.374 (df=2) | 0.830 |
| CAD severity | Altitude × diabetes | Global Wald χ²=0.058 (df=2) | 0.971 |

N=174 participants in 87 matched pairs. Models included altitude, diabetes, their interaction, age, sex, BMI, and hypertension, with variance clustered by matched pair. Effects are differences for untransformed outcomes, ratios of outcome+1 for transformed outcomes, and odds ratios for HRP. CAD severity entries are adjusted global multinomial tests from 500 matched-pair bootstrap replicates.

Supplementary Table S11. Components of the first MACE event

| **MACE component** | **n** | **%** |
| --- | --- | --- |
| All-cause death | 5 | 25.0 |
| Myocardial infarction | 2 | 10.0 |
| Ischemic stroke | 2 | 10.0 |
| Coronary revascularization | 11 | 55.0 |
| **Total** | **20** | **100.0** |

Values are n (%) of the 20 participants with MACE. MACE, major adverse cardiovascular events.

Supplementary Table S12. Baseline characteristics by follow-up status

| **Variable** | **Followed** | **Lost** | **P** |
| --- | --- | --- | --- |
| High-altitude residence, n (%) | 52 (43.7%) | 35 (63.6%) | 0.014 |
| HRP, n (%) | 75 (63.0%) | 44 (80.0%) | 0.025 |
| Male, n (%) | 68 (57.1%) | 41 (74.5%) | 0.027 |
| HDL-C, mmol/L | 1.15 ± 0.31 | 1.05 ± 0.29 | 0.046 |
| Smoking, n (%) | 25 (21.0%) | 19 (34.5%) | 0.056 |
| HbA1c, % | 6.84 ± 1.25 | 7.25 ± 1.72 | 0.257 |
| Total cholesterol, mmol/L | 4.15 ± 0.98 | 3.88 ± 1.12 | 0.125 |
| Plaque burden, % | 5.93 ± 5.23 | 7.56 ± 7.59 | 0.151 |
| Maximum stenosis, % | 57.85 ± 36.20 | 64.91 ± 31.22 | 0.191 |
| LDL-C, mmol/L | 2.48 ± 0.83 | 2.29 ± 0.94 | 0.218 |
| LAD-PCAT, HU | -76.95 ± 6.72 | -75.55 ± 7.34 | 0.231 |
| Age, years | 61.97 ± 10.97 | 64.16 ± 12.28 | 0.260 |
| Total plaque volume, mm³ | 72.94 [16.29-284.76] | 132.74 [43.91-359.57] | 0.153 |
| Triglycerides, mmol/L | 1.37 [0.98-1.93] | 1.34 [0.90-1.68] | 0.287 |
| LCX-PCAT, HU | -73.13 ± 8.33 | -72.11 ± 7.77 | 0.431 |
| Hemoglobin, g/L | 139.29 ± 19.96 | 141.53 ± 24.70 | 0.557 |
| Hypertension, n (%) | 62 (52.1%) | 26 (47.3%) | 0.554 |
| BMI, kg/m² | 26.03 ± 3.46 | 25.73 ± 3.66 | 0.610 |
| LCX CT-FFR | 0.91 ± 0.05 | 0.91 ± 0.05 | 0.636 |
| Diabetes, n (%) | 67 (56.3%) | 29 (52.7%) | 0.659 |
| Fasting blood glucose, mmol/L | 6.72 ± 2.51 | 6.51 ± 3.51 | 0.687 |
| Total CAC | 13.71 [0.00-156.45] | 30.20 [0.00-176.02] | 0.760 |
| LAD CT-FFR | 0.80 ± 0.09 | 0.80 ± 0.08 | 0.869 |
| RCA-PCAT, HU | -79.29 ± 9.77 | -79.11 ± 9.25 | 0.904 |
| RCA CT-FFR | 0.84 ± 0.11 | 0.84 ± 0.10 | 0.928 |

P values are descriptive and unadjusted. Pearson chi-square tests were used for categorical variables, Welch t tests for approximately normally distributed continuous variables, and Wilcoxon rank-sum tests for skewed continuous variables.

Supplementary Table S13. Firth penalized Cox sensitivity analysis

| **Variable** | **N** | **Events** | **ML HR** | **Low** | **High** | **P** | **Firth HR** | **Low** | **High** | **P** | **Conflict** |
| --- | --- | --- | --- | --- | --- | --- | --- | --- | --- | --- | --- |
| Diabetes (yes vs no) | 119 | 20 | 14.948 | 2 | 111.7 | 0.008 | 10.22 | 2.612 | 92.178 | <0.001 | 0 |
| HRP (present) | 119 | 20 | 14.938 | 1.994 | 111.9 | 0.008 | 10.226 | 2.602 | 92.395 | <0.001 | 0 |

Firth estimates were directionally consistent with the standard maximum-likelihood estimates. Penalization addresses quasi-complete separation but does not overcome sparse events, wide confidence intervals, or the absence of multivariable adjustment.

Supplementary Table S14. IPCW sensitivity analysis for differential follow-up

| **Variable** | **Unweighted HR** | **Low** | **High** | **P** | **IPCW HR** | **Low** | **High** | **P** |
| --- | --- | --- | --- | --- | --- | --- | --- | --- |
| Altitude (high vs low) | 0.771 | 0.324 | 1.836 | 0.557 | 0.739 | 0.304 | 1.797 | 0.505 |
| Diabetes (yes vs no) | 14.948 | 2.212 | 101.0 | 0.006 | 13.743 | 2.017 | 93.636 | 0.007 |
| HRP (present) | 14.938 | 1.987 | 112.3 | 0.009 | 17.022 | 2.248 | 128.9 | 0.006 |
| LAD-PCAT (per 5 HU) | 1.54 | 1.136 | 2.087 | 0.005 | 1.416 | 0.992 | 2.023 | 0.056 |
| CT-FFR LAD (per 0.05 decrease) | 1.431 | 1.241 | 1.649 | <0.001 | 1.375 | 1.172 | 1.612 | <0.001 |
| CAC (per log2[CAC+1]) | 1.25 | 1.079 | 1.449 | 0.003 | 1.238 | 1.062 | 1.443 | 0.006 |
| Total plaque volume (per 100 mm3) | 1.131 | 1.058 | 1.209 | <0.001 | 1.135 | 1.065 | 1.209 | <0.001 |
| Plaque burden (per 5 percentage points) | 1.67 | 1.264 | 2.206 | <0.001 | 1.639 | 1.236 | 2.174 | <0.001 |

N=119 followed; 55 lost; 20 events. Maximum IPCW was 1.964 and effective sample size was 111.9. The LAD-PCAT association retained direction but changed from P=0.005 to P=0.056.

Supplementary Table S15A. Apparent univariable Harrell C-indices

| **Variable** | **Harrell C** | **SE** | **Low** | **High** | **N** | **Events** | **Rank** |
| --- | --- | --- | --- | --- | --- | --- | --- |
| Total plaque volume (per 100 mm3) | 0.811 | 0.046 | 0.72 | 0.901 | 119 | 20 | 1 |
| CAD severity (per category) | 0.773 | 0.035 | 0.705 | 0.84 | 119 | 20 | 2 |
| CAC (per 100 Agatston units) | 0.739 | 0.062 | 0.618 | 0.86 | 119 | 20 | 3 |
| Plaque burden (per 5 percentage points) | 0.727 | 0.047 | 0.636 | 0.819 | 119 | 20 | 4 |
| Maximum stenosis (per 10 percentage points) | 0.715 | 0.045 | 0.626 | 0.804 | 119 | 20 | 5 |
| Diabetes (yes vs no) | 0.711 | 0.032 | 0.648 | 0.774 | 119 | 20 | 6 |
| CT-FFR LAD (per 0.05 decrease) | 0.689 | 0.073 | 0.546 | 0.833 | 119 | 20 | 7 |
| HRP (present) | 0.687 | 0.029 | 0.63 | 0.744 | 119 | 20 | 8 |
| RCA-PCAT (per 5 HU increase) | 0.669 | 0.066 | 0.538 | 0.799 | 119 | 20 | 9 |
| CT-FFR RCA (per 0.05 decrease) | 0.663 | 0.056 | 0.553 | 0.774 | 119 | 20 | 10 |
| LCX-PCAT (per 5 HU increase) | 0.657 | 0.067 | 0.525 | 0.789 | 119 | 20 | 11 |
| Age (per 10 years) | 0.639 | 0.072 | 0.497 | 0.78 | 119 | 20 | 12 |
| Smoking | 0.61 | 0.055 | 0.503 | 0.718 | 119 | 20 | 13 |
| LAD-PCAT (per 5 HU increase) | 0.609 | 0.076 | 0.459 | 0.759 | 119 | 20 | 14 |
| CT-FFR LCX (per 0.05 decrease) | 0.609 | 0.075 | 0.461 | 0.756 | 119 | 20 | 15 |
| Sex (male vs female) | 0.565 | 0.053 | 0.461 | 0.669 | 119 | 20 | 16 |
| Altitude (high vs low) | 0.507 | 0.06 | 0.389 | 0.624 | 119 | 20 | 17 |
| Hypertension | 0.495 | 0.068 | 0.361 | 0.629 | 119 | 20 | 18 |

C-indices are apparent, univariable discrimination estimates for censored outcomes (N=119; events=20); they are not validated clinical performance estimates and no threshold was derived.

Supplementary Table S15B. Proportional-hazards diagnostics

| **Variable** | **Covariate χ²** | **P** | **Global χ²** | **Global P** | **BH q** | **Violation** |
| --- | --- | --- | --- | --- | --- | --- |
| Altitude (high vs low) | 0.608 | 0.435 | 0.608 | 0.435 | 0.916 | No |
| Diabetes (yes vs no) | 1.317 | 0.251 | 1.317 | 0.251 | 0.916 | No |
| Age (per 10 years) | 0.467 | 0.494 | 0.467 | 0.494 | 0.916 | No |
| Sex (male vs female) | 0.663 | 0.415 | 0.663 | 0.415 | 0.916 | No |
| Hypertension | 0.234 | 0.629 | 0.234 | 0.629 | 0.916 | No |
| Smoking | 0.716 | 0.397 | 0.716 | 0.397 | 0.916 | No |
| HRP (present) | 0.075 | 0.784 | 0.075 | 0.784 | 0.940 | No |
| LAD-PCAT (per 5 HU increase) | 1.46 | 0.227 | 1.46 | 0.227 | 0.916 | No |
| LCX-PCAT (per 5 HU increase) | 0.303 | 0.582 | 0.303 | 0.582 | 0.916 | No |
| RCA-PCAT (per 5 HU increase) | 0.013 | 0.909 | 0.013 | 0.909 | 0.959 | No |
| CT-FFR LAD (per 0.05 decrease) | 0.003 | 0.959 | 0.003 | 0.959 | 0.959 | No |
| CT-FFR LCX (per 0.05 decrease) | 1.239 | 0.266 | 1.239 | 0.266 | 0.916 | No |
| CT-FFR RCA (per 0.05 decrease) | 0.546 | 0.460 | 0.546 | 0.460 | 0.916 | No |
| CAC (per 100 Agatston units) | 0.192 | 0.661 | 0.192 | 0.661 | 0.916 | No |
| Total plaque volume (per 100 mm3) | 0.127 | 0.721 | 0.127 | 0.721 | 0.927 | No |
| Plaque burden (per 5 percentage points) | 0.886 | 0.347 | 0.886 | 0.347 | 0.916 | No |
| Maximum stenosis (per 10 percentage points) | 0.222 | 0.638 | 0.222 | 0.638 | 0.916 | No |
| CAD severity (per category) | 0.042 | 0.838 | 0.042 | 0.838 | 0.943 | No |

All 18 pair-cluster-robust univariable Cox models were assessed using scaled Schoenfeld residuals (cox.zph with the Kaplan–Meier time transformation). Global P values ranged from 0.227 to 0.959; none was <0.05.

**Supplementary Figure S1**

Kernel-density distributions of estimated propensity scores in the 248-participant source cohort before matching, shown separately by diabetes stratum and altitude group. Greater overlap indicates greater common support for matching. DM, diabetes mellitus; non-DM, no diabetes; PSM, propensity score matching.

**Supplementary Figure S2**

Standardized mean differences for the four matching variables before and after matching, shown separately within the T2DM and non-T2DM strata. Dashed lines mark SMD=−0.10 and +0.10. Matching used age, sex, BMI, and hypertension. BMI, body mass index; SMD, standardized mean difference; T2DM, type 2 diabetes mellitus.

**Supplementary Figure S3**

Apparent univariable Harrell C-indices with 95% confidence intervals for MACE in the 119 participants with follow-up (20 events). Values summarize in-sample discrimination for censored outcomes and were not internally or externally validated; no clinical threshold was derived. CAC, coronary artery calcium; CAD, coronary artery disease; CI, confidence interval; CT-FFR, CCTA-derived fractional flow reserve; HRP, high-risk plaque; LAD, left anterior descending artery; LCX, left circumflex artery; MACE, major adverse cardiovascular events; PCAT, pericoronary adipose tissue attenuation; RCA, right coronary artery.
